# Exploring influential nodes using global and local information

**DOI:** 10.1038/s41598-022-26984-4

**Published:** 2022-12-29

**Authors:** Haifeng Hu, Zejun Sun, Feifei Wang, Liwen Zhang, Guan Wang

**Affiliations:** Pingdingshan University, Pingdingshan, 467000 China

**Keywords:** Computational science, Computer science

## Abstract

In complex networks, key nodes are important factors that directly affect network structure and functions. Therefore, accurate mining and identification of key nodes are crucial to achieving better control and a higher utilization rate of complex networks. To address this problem, this paper proposes an accurate and efficient algorithm for critical node mining. The influential nodes are determined using both global and local information (GLI) to solve the shortcoming of the existing key node identification methods that consider either local or global information. The proposed method considers two main factors, global and local influences. The global influence is determined using the K-shell hierarchical information of a node, and local influence is obtained considering the number of edges connected by the node and the given values of adjacent nodes. The given values of adjacent nodes are determined based on the degree and K-shell hierarchical information. Further, the similarity coefficient of neighbors is considered, which enhances the differentiation degree of the adjacent given values. The proposed method solves the problems of the high complexity of global information-based algorithms and the low accuracy of local information-based algorithms. The proposed method is verified by simulation experiments using the SIR and SI models as a reference, and twelve typical real-world networks are used for the comparison. The proposed GLI algorithm is compared with several common algorithms at different periods. The comparison results show that the GLI algorithm can effectively explore influential nodes in complex networks.

## Introduction

In recent years, human production and life have become increasingly inseparable from different types of networks^1^. In sensor networks, a sensor represents the main device to obtain desired information. Then, through a self-group sensor network, the perceived information can be transmitted to a server for further data processing^2,3^. Financial networks have brought great convenience to human life. For instance, when people shop on the Internet, they can enjoy the convenience brought by the consumer financial network^4^. In addition, in social networks, people can make new friends, thus constantly expanding their personal connections and achieving better communication with other people^5^. Currently, many phenomena can be described using complex networks, such as social activities, smart sensor applications, consumer finance, and consumer finance services^6^. Complex networks are associated with countless nodes, so accurately exploring and identifying their key nodes can solve many application problems, including fault node positioning, prevention and control of fraud risk, and friend recommendations^7–9^. Therefore, how to design an efficient algorithm to identify key nodes of a complex network accurately is an urgent problem. In an intelligent sensor network, accurately identifying and effectively protecting important sensors can avoid security attacks^10^. In this way, when a fault occurs in a network, the fault position can be located fast and the fault can be timely addressed^11^. Further, in a consumer finance business network, the risk of fraud of credit customers can be judged effectively^12^, financial institutions can be helped to filter inferior users, and the first risk control link can be established to delete fraud personnel^13^. In addition, when recommending friends in a community network, the identification of key nodes can help users to recommend friends that they have not added yet but may know and send the result to users as a "friend recommendation," which can improve user loyalty^14^.

The most popular approach to explore influential nodes in complex networks has been to used centrality measures^15–21^. From the perspective of global and local information, typical methods based on local information include methods considering the degree centrality (DC)^22^ and methods considering the eigenvector centrality (EC)^23^. The DC-based methods calculate the node influence by analyzing the number of edges connected by nodes, which has the advantages of high simplicity and fast calculation speed. However, these methods consider only local information and have low accuracy^24^. The EC-based method calculates the node influence considering both the number and the information of adjacent nodes. Namely, when the number of adjacent nodes is very large, their influence will be very strong. But The EC method score much prefers to concentrate in a few nodes under common conditions, making it hard to distinguish among the nodes^25^. In contrast, methods based on global information include the closeness centrality (CC)^26^, betweenness centrality (BC)^27^, and K-shell decomposition methods^28^. The BC and CC methods have high accuracy in identifying critical nodes, but their long computational time limits their application to large networks^29^. The K-shell decomposition method calculates the node influence based on the node location, but the hierarchical results are coarse-grained, resulting in a low node discrimination^30^. The aforementioned algorithms consider either global or local information of nodes, which causes certain limitations in practical applications.

The node influence information obtained based on multiple datasets is more accurate than that obtained using only a single attribute^31^. Therefore, this study uses global information to calculate the global influence of nodes but local information to calculate their own influence and the influence of their adjacent nodes. The nodes with the same global influence are distinguished to improve the differentiation effect of node influence.

### Basic ideas of proposed GLI method

The factors should be considered comprehensively from both global and local aspects. For instance, an important project in the real world can usually be decomposed into multiple subprojects, so a team responsible for completing the project needs to be divided into groups, each of which will be responsible for one subproject. Such a team can be regarded as a complex network where team members denote nodes, and the influence of each member depends on his position in the network. The higher the position is, the greater the number of resources will be; for instance, the project team leader is more influential than the group leader, and the group leader is more influential than the ordinary members. At the same time, the self-capability and the help provided by the team members are also important factors that define the influence. If members A and B have many common neighbors, member B is considered to be close to member A, members B and A have a great similarity, and member B has a greater influence on member A. The more similar the other members are with a particular member, the greater their influence on the member is. Accordingly, the proposed GLI method considers the contribution of both global and local information. First, global information is represented through network hierarchy obtained by the K-shell method; next, local information is represented by the degree of self and adjacent nodes, and adjacent nodes’ Ks values. In addition, the similarity coefficient is introduced, and the higher the similarity between the nodes is, the greater the contribution provided by the adjacent nodes will be.

### Contribution of proposed GLI

This study provides an innovative research perspective for the identification of key nodes in a complex network. The proposed GLI algorithm’s innovation is mainly reflected in three aspects, which are as follows:

1. An accurate key node identification method, which considers the influence of nodes from both global and local aspects, is proposed. The shortcomings of the existing coarse-grained methods based on global information are addressed, and the accuracy of the key node identification is improved;
2. The proposed GLI method uses the similarity coefficient between nodes in a network, which enhances the differentiation ability of adjacent gives values and improves the identification ability of key nodes;
3. The proposed GLI method considers both global and local information, which makes it highly practical and suitable for large and complex networks.

The rest of this article is organized as follows. Section “Related work” describes related work. Section “Proposed GLI” describes the proposed GLI method and presents its design idea and specific working process. Section “Experimental Results” compares the proposed GLI method and classical algorithm on different datasets and analyzes the comparison results. Finally, Section “Conclusion” summarizes the main contributions of the study.

## Related work

Many factors influence the accuracy of key node identification in complex networks. In the following, a very brief survey of the methods relevant to the proposed GLI is provided. The measures considered in these methods are introduced from the global and local perspectives.

1. Local centrality
  DC: This is a local centrality measure related to the number of edges connected by nodes.
  EC: This is a local centrality measure related to a node’s degree and influence of its neighbors.
  PageRank^32^(PR): Similar to the EC, this measure reflects the importance of a node, which is determined by both the quantity and the quality of adjacent nodes. This is a local centrality measure using the node degree and the PR value of the neighboring nodes.
  ProfitLeader^33^(PL): The ProfitLeader algorithm computes the profit a node provides to the other nodes, where the importance of the node is related to the profit. This is a local centrality measure using node profit and sharing probability to its neighbors.
2. Global centrality
  BC: Global centrality measure uses the number of shortest paths through the node.
  CC: Global centrality measure denotes the relative shortest path between the pairs of nodes.
  K-shell: In a network, nodes are decomposed in a layer-by-layer manner according to their degree values, and the node importance is evaluated from the level where the node is located in. This is a global centrality measure based on the node degree.
3. Combination of global and local centrality
  GIN^34^: This is a combination of global and local centrality obtained based on the distance, degree, and all other node-related information.
  KBKNR^35^: The KBKNR algorithm reflects the influence of adjacent nodes and secondary-adjacent nodes through the influence coefficient, which is then used to distinguish the nodes based on their importance. This is a combination of global and local centrality using the k-shell, distance, and degree values.
  RLGI^36^: This is a combination of global and local centrality obtained based on the k-shell and degree.
  GLS^37^: Combination of global and local centrality based on the k-shell, distance, degree, and eigenvector centrality.

## Proposed GLI

### Definition

The research objectives of this paper include undirected and unweighted networks, which can be expressed as *Graph* = (*Vertex*, *Edge*). This section introduces some of the definitions. The following definitions are related to an undirected, unweighted network.

#### Definition 1

Node degree^38^: The node degree represents the number of edges that a particular node joins. Assume, a network is defined as *A* = (*a*_*i*j_) *N* × *N*; then, the node degree *d*(*v*_*i*_) can be expressed as follows:

1 $$ d\left( {vi} \right) = \sum\limits_{j = 1}^{N} {aij} = \sum\limits_{j = 1}^{N} {aji} $$

2 $$ \max D = \max \left( {d\left( {vi} \right)} \right) $$

where max*D* indicates the maximal node degree.

#### Definition 2

K-shell value of a node: The K-shell value is obtained by the K-shell algorithm, and it is calculated by:

3 $$ Ks\left( {vi} \right) = K - shell\left( G \right) $$

#### Definition 3

Global node influence: The K-shell algorithm can calculate the global position value of a node *v*_*i*_, which measures the global influence of *v*_*i*_, *Global*(*v*_*i*_). The specific calculation formula is as follows:

4 $$ Global\left( {vi} \right) = Ks\left( {vi} \right) $$

#### Definition 4

Adjacent node similarity: A given value of an adjacent node represents the factor affecting the importance of a node, and there is a relationship between the given value size and the similarity between the nodes. Adjacent nodes with different local structures may enhance the differentiation effect of a given influence of adjacent nodes. In GLI method, the higher the node similarity is, the higher the proportion of influence the node can provide, so the proposed algorithm adopts the Jaccard similarity coefficient^39^J(*v*_*i*_,*v*_*j*_), which reflects the similarity of adjacent nodes, and it is calculated by:

5 $$ Jacc\left( {vi,vj} \right) = \left| {\frac{{n\left( {vi} \right) \cap n\left( {vj} \right)}}{{n\left( {vi} \right) \cup n\left( {vj} \right)}}} \right| $$

where *n*(*v*_*i*_) represents the set of nodes that have common edges with a node $vi$ and contains this node, *n*(*v*_*j*_) denotes the set of nodes that have common edges with node $vj$ and contains node *v*_*j*_.

#### Definition 5

Local influence: Local influence considers two factors: personal influence of a node and the contribution of its adjacent nodes.

Assume that *P*(*v*_*i*_) denotes the personal influence of a node; then, *P*(*v*_*i*_) is determined by *d*(*v*_*i*_) and calculated by:

6 $$ P\left( {vi} \right) = d\left( {vi} \right) $$

Assume that *v*_*j*_ is an adjacent node of node *v*_*i*_; then, the given value *П*(*v*_*j*_) of node *v*_*j*_ can be obtained by comprehensively considering *d*(*v*_*i*_), *Ks*(*v*_*j*_), and *J*(*v*_*i*_,*v*_*j*_), which is expressed by:

7 $$ \prod {\left( {vj} \right)} = d\left( {vj} \right) \times Jacc\left( {vi,vj} \right) + Ks\left( {vj} \right) $$

The *Sum*(*v*_*i*_) represents the sum of the given influence for all the adjacent nodes, *Sum*(*v*_*i*_) calculation formula is as follows:

8 $$ Sum\left( {vi} \right) = \sum\limits_{{vj \in \Gamma \left( {vi} \right)}} {\prod {\left( {vj} \right)} } $$

Further, assume that in the considered network, node *v*_*i*_ has the most max*D* adjacent nodes to provide the given influence. Then, *Sum*(*v*_*i*_) is divided by max*D* to normalize, and the given influence of node *vi* on its adjacent node is obtained by:

9 $$ N\left( {vi} \right) = {{Sum\left( {vi} \right)} \mathord{\left/ {\vphantom {{Sum\left( {vi} \right)} {\max D}}} \right. \kern-0pt} {\max D}} $$

where *Local*(*v*_*i*_) indicates local influence, and it is defined by:

10 $$ Local\left( {vi} \right) = P\left( {vi} \right) + N\left( {vi} \right) $$

#### Definition 6

Node influence: The node influence *I*(*v*_*i*_) depends on *Global*(*v*_*i*_) and *Local*(*v*_*i*_), and it is calculated as follows:

11 $$ I\left( {vi} \right) = Local\left( {vi} \right) + Global\left( {vi} \right). $$

### Proposed model evaluation

To verify the effect of the proposed algorithm, two evaluation models, the SIR and SI models, were selected. The reliability of the comparison analysis was validated by experimental results.

#### SIR model

The SIR model^40^ is a mathematical model describing disease transmission, which is a general standard for evaluating the accuracy of node identification.

In the SIR model, network nodes are divided into three categories as follows:

- Susceptible: A susceptible node is a node that is not sick but lacks the immune ability and is vulnerable to the infection after contact with a sick node.
- Infection: A sick node is an infected node that can infect a susceptible node.
- Removed: A node removed from a network, recovered (with immunity) or dead; these nodes are no longer involved in the infection process.

Next, assume that at time *t*, the total node number *All*(*t*) is unchanged, and nodes can be in one of the three states: susceptible to infection point *Susceptible*(*t*), sick node *Infection*(*t*), or removed from node *Removed*(*t*), and it holds that *All*(*t*) = *Susceptible*(*t*) + *Infection*(*t*) + *Removed*(*t*).

At time *t*, the number of infected nodes is *α* * *Susceptible*(*t*) * *Infection*(*t*).

After the time interval of *Δt*, changes in the numbers of susceptible, infection, and removed nodes are respectively as follows:

- Susceptible: *Susceptible*(*Δt*) =  − α * Susceptible(*t*) * Infection(*t*).
- Infection: *Infection*(*Δt*) = α * Susceptible(*t*) * Infection(*t*) − β * Infection(*t*).
- Removed: *Removed*(*Δt*) = β * Infection( *t* ).

#### SI model

Similar to the SIR model, the SI model^41^ is the simplest disease transmission model. In the SI model, network nodes are divided into two groups: susceptible nodes and infection nodes. At time *t*, a susceptible node may become an infected node with a probability of *α*, and this process is irreversible.

In the experiment conducted in this study, network nodes were treated as sick nodes, and the number of infected nodes was calculated using the SIR and SI models. The calculation process included multiple iterations to illustrate the infection influence of nodes. The results of the network obtained by the above two models were used as an evaluation criterion. The results of the proposed GLI algorithm and several related algorithms were compared.

### Kendall coefficient

The Kendall coefficient *τ* is used in this study to determine the similarity between the ranking results of the proposed algorithm and those of the SIR model^42^ on the same network. Assume nodes *v*_*i*_ and *v*_*j*_ are selected by the GLI algorithm to obtain the values *GLI*(*v*_*i*_) and *GLI*(*v*_*j*_). Then, node *v*_*i*_ and *v*_*j*_ are processed by the SIR model, and values *SIR*(*v*_*i*_) and *SIR*(*v*_*j*_) are obtained. If *GLI*(*v*_*i*_) > *GLI*(*v*_*j*_) and *SIR*(*v*_*i*_) > *SIR*(v_*j*_), or *GLI*(*v*_*i*_) < *GLI*(v_*j*_) and *SIR*(*v*_*i*_) < *SIR*(*v*_*j*_), then the resulting values are considered consistent, and $\tau$ = 1. If *GLI*(*v*_*i*_) > *GLI*(*v*_*j*_) and *SIR*(*v*_*i*_) < *SIR*(*v*_*j*_), or *GLI*(*v*_*i*_) < *GLI*(*v*_*j*_) and *SIR*(*v*_*i*_) > *SIR*(*v*_*j*_), then the resulting values are considered inconsistent, and $\tau$ = -1. The specific calculation formula is as follows:

12 $$ \tau (X,Y) = \frac{{2 * \left( {n_{c} - n_{d} } \right)}}{{n * \left( {n - 1} \right)}} $$

where *X* and *Y* represent the evaluated object, *n*_*c*_ is the number of consistencies in two sequences, and *n*_*d*_ indicates the number of inconsistencies in the two sequences.

## Experimental results

### Algorithm process


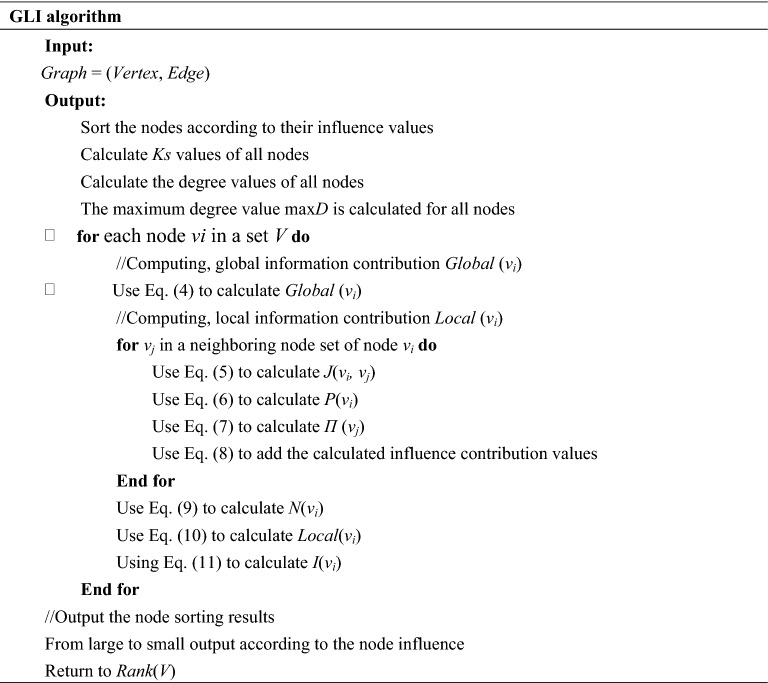


For detailed procedures, please refer to Supplementary Information.

## Example description

**Figure 1:**
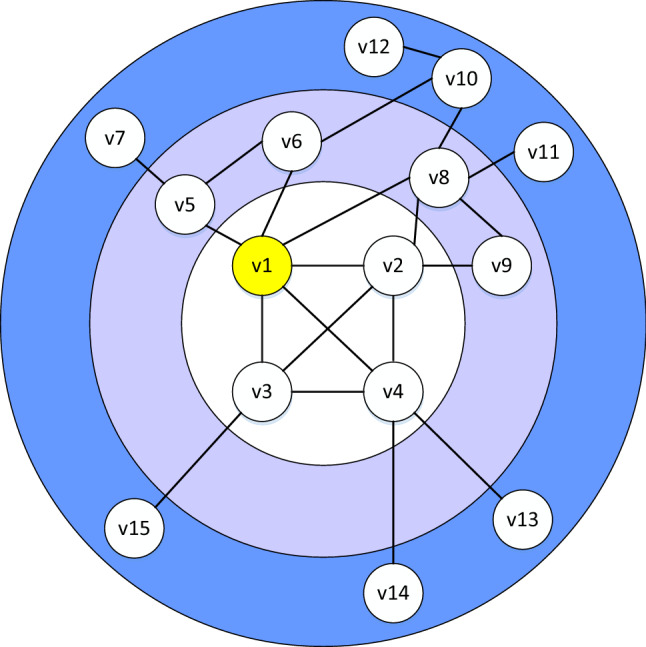
A network diagram, where yellow nodes indicate the sample nodes. The K-shell algorithm is used to divide the network into three layers, and node *v*_1_ is in the third layer.

In Fig. 1, the proposed algorithm is described in the example of the calculation process of the influence of node *v*_*1*_:

1. Node degree

According to the idea of GLI algorithm, the node degree of node *v*_*1*_ and its adjacent nodes is calculated by Eq. (1), and the results are shown in Table 1. The maximum degree is max*D* = 6, and it is calculated by Eq. (2).

**Table 1:** Degree of *v*_*1*_ and adjacent nodes.

Node	*v*_*1*_	*v*_*2*_	*v*_*3*_	*v*_*4*_	*v*_*5*_	*v*_*6*_	*v*_*8*_
Degree	6	5	4	5	3	2	5

(2) *Ks* value of nodes

According to Eq. (3), after the decomposition by the K-shell algorithm, the *Ks* value of node *v*_*1*_ and its adjacent nodes is obtained, and the results are shown in Table 2.

**Table 2:** Ks of *v*_*1*_ and adjacent nodes.

Node	*v*_*1*_	*v*_*2*_	*v*_*3*_	*v*_*4*_	*v*_*5*_	*v*_*6*_	*v*_*8*_
Ks	3	3	3	3	2	2	2

(3) Global influence of node

According to Eq. (4), it is obtained that: *Global* (*v*_*1*_) = *Ks*(*v*_*1*_) = 3.

(4) Similarity coefficient of nodes

According to Eq. (5), the similarity coefficient of node *v*_*1*_ and its neighbors, denoted by *J*(*v*_*1*_,*v*_*j*_), is calculated, and the obtained results are shown in Table 3.

**Table 3:** The similarity coefficient values of node *v*_*1*_ and its adjacent nodes.

*Jacc*(*v*_*1*_,*v*_*2*_)	*Jacc*(*v*_*1*_,*v*_*3*_)	*Jacc*(*v*_*1*_,*v*_*4*_)	*Jacc*(*v*_*1*_,*v*_*5*_)	*Jacc*(*v*_*1*_,*v*_*6*_)	*Jacc*(*v*_*1*_,*v*_*8*_)
0.625	0.5	0.444444	0.375	0.4285714	0.3

(5) Local influence

The given influence of the adjacent nodes of node *v*_*1*_ is calculated by Eq. (7), and the results are shown in Table 4.

**Table 4:** Local influence values of node *v*_*1*_ and its adjacent nodes.

*П*(*v*_*2*_)	*П*(*v*_*3*_)	*П*(*v*_*4*_)	*П*(*v*_*5*_)	*П*(*v*_*6*_)	*П*(*v*_*8*_)
6.125	5.0	5.222222	3.125	2.857143	3.5

Nodes *v*_*2*_ and *v*_*4*_ have the same degree and Ks values, but different similarity coefficient values, so their contribution values to node *v*_*i*_ differ. Thus, it improves the discrimination degree values of the neighboring nodes.

Second, according to Eq. (6), it holds that: *P*(*v*_*1*_) = *d*(*v*_*1*_) = 6.

Finally, according to Eq. (10), we have *Local*(*v*_*1*_) = 10.304894.

(6) Node influence

According to Eq. (11), it can be obtained that: *I(v*_*1*_*)* = *Local(v*_*1*_*)* + *Global(v*_*1*_*)* = 13.304894.

Following the above-presented steps, the influence values of all nodes in Fig. 1 are calculated, as shown in Table 5.

**Table 5:** Influence of the nodes in the sample network presented in Fig. 1.

Node	*v*_*1*_	*v*_*2*_	*v*_*4*_	*v*_*3*_	*v*_*8*_	*v*_*5*_	*v*_*9*_	*v*_*6*_	*v*_*10*_	*v*_*13*_	*v*_*14*_	*v*_*15*_	*v*_*11*_	*v*_*7*_	*v*_*12*_
*I*	13.30	12.17	11.18	10.18	9.70	6.71	5.67	5.54	3.85	2.78	2.78	2.77	2.61	2.58	2.39

### Data description

Twelve real representative networks were selected to evaluate the proposed GLI algorithm, and they are as follows:

1. Blogs network^43^: This is a network of hyperlinks between the homepage of the 2004 US election blog, which includes 1,224 nodes and 19,052 edges.
2. Ca-Astroph network^44^: This is a collaborative network of scientific collaboration relationships between the astrophysical category author papers; it includes 18,771 nodes and 198,050 edges.
3. Friendship network^45^: This network represents the connections between users on hamsterster.com and has 1,858 nodes and 12,534 edges.
4. Email EU32430 network^46^: This network expresses the communication between email users and consists of 32,430 nodes and 54,397 edges.
5. Polbooks network^47^: A network of online book sales built on the relationships between American political book buyers, with 105 nodes and 441 edges.
6. Jazz network^48^: A collaboration network of a group of jazz musicians, including 198 nodes and 2,742 edges.
7. Football network^33^: This network represents a college football league and consists of 115 nodes and 616 edges.
8. Karate network^49^: This network is a network of Karate Club members, with 34 nodes and 78 edges.
9. Protein network^50^: This network consists of proteins that interact with each other, and it includes 1,870 nodes and 2,277 edges.
10. USAir2010 network^51^: The 2010 US network contains 1,574 nodes and 17,215 edges.
11. Reactome network^52^: This network consists of proteins that interact with each other, and it includes 6,327 nodes and 147,547 edges.
12. Brightkite network^53^: This network is a social networking, and it includes 58,228 nodes and 214,078 edges.

The relevant property statistics of the experimental datasets are given in Table 6.

**Table 6:** Characteristic statistics for ten real networks used in the comparison experiment.

Network	|V|	|E|	< avgD >	max*D*	< C >
Blogs	1224	19,025	31.087	351	0.3197
Ca-Astroph	18,771	198,050	21.102	236	0.677
Friendships	1858	12,534	13.492	272	0.167
EmailEU32430	32,430	54,397	3.355	623	0.1127
Polbooks	105	441	8.4	25	0.488
Jazz	198	2742	27.697	100	0.633
Football	115	616	10.6	12	0.403
Karate	34	78	4.588	17	0.588
Protein	1870	2277	2.435	56	0.171
USAir2010	1574	17,215	21.78	314	0.637
Reactome	6327	147,547	46.64	855	0.67
Brightkite	58,228	214,078	7.353	1134	0.271

### Experimental result

To evaluate the applicability of the GLI algorithm, nine typical algorithms were implemented by Python, and the experiments were performed on ten datasets of different sizes. The experimental hardware platform included a Lenovo desktop computer, a CPU: i5-10,100, a memory of 32 GB; the software environment was Spyder (Python 3.7.3).

#### Experimental results comparison with the SIR model

1. Kendall value analysis

**Figure 2:**
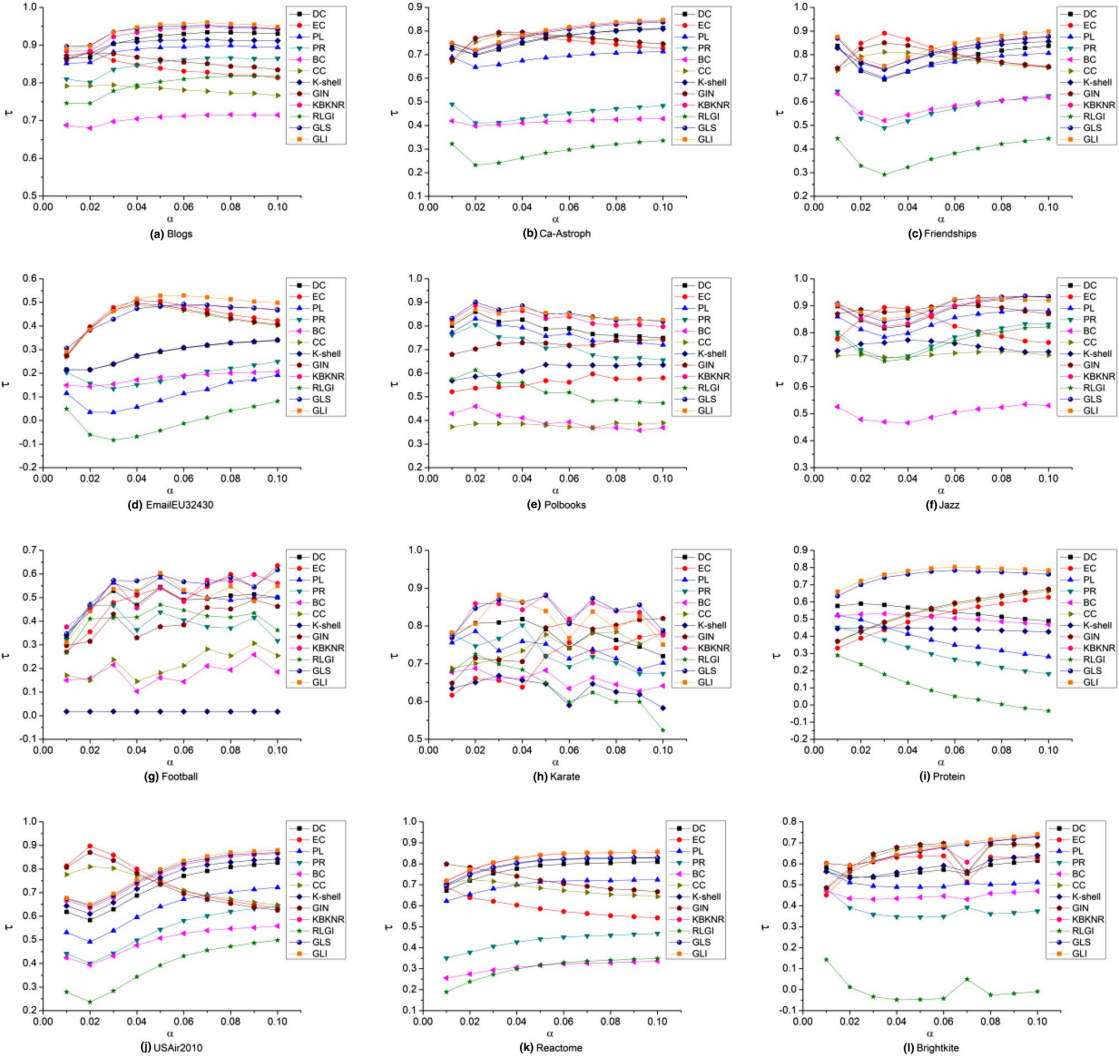
Kendall τ values of different algorithms compared with the SIR model result on 12 networks. Infection probability *α* varied from 0.01 to 0.1.

The experimental networks and their characteristic information are shown in Table 6. The range of *α* in the SIR model was [0.01, 0.1], and the step length was 0.01; this value was selected to avoid the network infection being too slow or too fast. First, the Kendall value results of the BC, CC, DC, EC, k-shell, PR, GIN, PL, KBKNR, RLGI, GLS, and GLI algorithms on different datasets were compared with the infection results of the SIR model. Then, the Kendall values of the proposed GLI algorithm and the other algorithms were compared. The comparison results are shown in Fig. 2.

The Kendall *τ* values of the GLI was the highest at all infection probabilities in the Protein network. When calculating the influence coefficient, the KBKNR algorithm takes the number of neighbor nodes as the divisor. While in the protein network, for nodes with a presence degree of zero, the algorithm cannot run correctly. In the six infection networks, the Blogs, Ca-Astroph, Friendships, EmailEU32430, Reactome and USAir2010 networks, the GLI algorithm performed better than the other algorithms. Among the above seven networks, the values of the maximum degree is relatively large, the values of the average degree was relatively small, and the distinction degree of the nodes’ degree values was large. Therefore, GLI has a better performance in these networks.

In the Brightkite network, the GLI, GIN, and KBKNR algorithms were superior to the other algorithms. In the Polbooks and Karate networks, the Kendall *τ* value of the GLS algorithm was higher than that of the GLI algorithm, but the GLI algorithm performed better than the other algorithms. In Brightkite, Polbooks and Karate networks, the distinction degree of the nodes’ degree values was small. Therefore, the advantages of the GLI algorithm are not obvious.

There are strong relationships between the hierarchical measure, the centrality measure, and the topological properties of the network^54^. In Jazz and football networks, the connections between local nodes are relatively dense and have an obvious community structure. The distinction between K-shell value and degree value is not high. Therefore, the GLI algorithm does not work well in these two networks.

(2) Optimal algorithm under different infection probabilities

**Figure 3:**
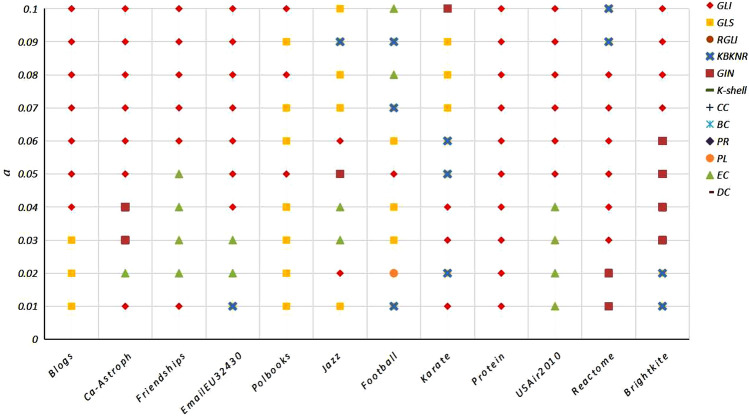
The maximum Kendall τ value results of the algorithms for different infection probabilities; the abscissa represents the network datasets, the ordinate indicates the infection probability α. Data presented in Fig. 3 correspond to the algorithm of the maximum Kendall τ values.

As illustrated in Fig. 3, the GLI algorithm achieved a maximum value of 51.67% at different infection probabilities on the 12 networks. The maximum results of the other algorithms were as follows: 16.67% for the GLS algorithm, 10% for the KBKNR algorithm, 12.5% for the EC algorithm, 8.33% for the GIN algorithm, and 0.83% for the PL algorithm. In addition, the GLI algorithm performed well on all networks.

(3) Top-15 important nodes in different networks

Without a loss of generality, in this experiment, the SIR model infection probability α was set to 0.02, and the recovery probability was set to one. First, the results of different algorithms on the network datasets were obtained and compared with the SIR model. Then, the 15 most influential nodes were extracted from the results. Finally, the algorithms’ performances were analyzed by ranking the nodes. The first 15 nodes of the Karate, Jazz, and Ca-Astroph networks in the large, medium, and small three-type networks were selected and illustrated.

As presented in Table 7, the GLI, EC, GLS and PR algorithms achieved identical results for 14 nodes out of the first 15 nodes of the SIR model. The GLI, GLS and PR algorithms were rank-aligned with the top-three nodes of the SIR model, achieving the best results among all algorithms. The EC algorithm ranked the first two nodes of the SIR correctly and was the second-best performing algorithm, following the GLI, GLS and PR algorithms.

**Table 7:** Top-15 nodes in the Karate network obtained by different algorithms.

DC	EC	PL	PR	BC	CC	K-shell	GIN	KBKNR	RLGI	GLS	GLI	SIR
34	34	1	34	1	1	1	1	1	1	34	34	34
1	1	34	1	34	3	2	34	34	34	1	1	1
33	3	33	33	33	34	3	3	33	33	33	33	33
3	33	3	3	3	32	4	33	3	3	3	3	2
2	2	2	2	32	9	8	2	2	2	2	2	3
4	9	32	32	9	14	9	32	4	4	32	4	32
32	14	9	4	2	33	14	9	9	24	4	14	14
9	4	14	24	14	20	31	14	14	25	9	9	4
14	32	4	9	20	2	33	4	31	32	14	32	9
24	31	24	14	6	4	34	20	8	26	24	8	24
6	8	6	7	7	31	5	31	32	9	31	24	30
7	24	7	6	28	28	6	28	24	14	8	31	31
8	20	28	30	24	29	7	8	28	6	30	30	8
31	30	31	28	31	8	11	29	30	7	28	6	28
28	28	30	31	4	10	20	24	6	28	7	7	6

Table 8 shows that the top-15 nodes of the Jazz network were ranked, and the PL algorithms achieved the best results. Fourteen nodes out of 15 nodes were the same as those of the SIR model, and the ranking of the first four nodes was completely consistent with the SIR model. The GLI algorithm ranked 12 nodes out of the 15 nodes the same as in the SIR model, and the first four nodes were identical to the first four nodes of the SIR model. Although the GLI algorithm performed poorly compared with the PL algorithm, it achieved better results than the other algorithms, which indicated that the GLI algorithm was effective.

**Table 8:** Top-15 nodes in the Jazz network obtained by different algorithms.

DC	EC	PL	PR	BC	CC	K-shell	GIN	KBKNR	RLGI	GLS	GLI	SIR
135	59	59	135	135	135	34	135	59	135	59	59	59
59	131	135	59	152	59	59	59	167	59	131	135	135
131	135	131	167	59	167	97	131	131	167	135	131	131
167	167	167	131	148	69	98	167	98	148	167	167	167
69	107	107	148	167	82	99	69	107	95	107	107	98
98	98	69	69	166	131	100	107	121	131	98	98	69
107	130	98	166	188	193	107	98	130	166	130	130	107
82	69	130	82	114	121	130	82	134	69	69	121	82
157	82	193	95	95	173	131	193	99	152	100	69	193
6	193	6	4	82	157	153	157	100	4	121	99	6
130	121	157	157	4	98	167	130	104	6	99	100	130
193	99	82	152	69	191	121	121	97	82	157	82	99
121	100	191	98	134	107	109	6	178	157	6	157	157
191	97	121	107	121	68	31	191	109	191	34	134	163
148	157	163	163	173	148	134	68	34	134	82	6	191

As shown in Table 9, for the first 15 nodes of the Ca-Astroph network, 13 nodes of the GLI, DC, and GIN algorithms were the same as those in the SIR model, and the importance of the first 15 nodes was basically the same. However, the GLI and GIN algorithms had the same two nodes as the SIR, GLI, and GIN algorithms, which achieved the best results and were followed by the DC algorithm. For the PR and CC algorithms, 12 nodes and 11 nodes out of 15 nodes were identical to those in the SIR model, respectively. For the Ca-Astroph network, the worst-performing algorithms were the K-shell and KBKNR, having only one node identical to the SIR model nodes.

**Table 9:** Top-15 nodes in the Ca-Astroph network obtained by different algorithms.

DC	EC	PL	PR	BC	CC	K-shell	GIN	KBKNR	RLGI	GLS	GLI	SIR
326	326	326	326	256	2295	65	326	967	326	326	326	124
299	304	2126	256	877	326	983	2126	956	299	285	2295	326
304	2295	2295	299	3749	256	906	967	919	2295	304	304	2124
2295	2126	304	304	1237	967	939	2295	360	256	124	299	304
2126	2124	967	2295	299	299	954	304	991	1091	165	2126	2295
967	285	299	967	326	304	967	2124	906	304	117	967	299
285	124	165	1091	360	2126	901	285	988	15,743	2124	285	285
256	2299	124	2126	967	124	483	299	901	967	2126	2124	2126
2124	165	2124	3749	4309	165	259	165	259	285	2295	877	967
877	2277	256	2285	202	117	360	877	939	8848	1129	256	2277
124	117	117	285	1091	1129	376	2277	963	3749	126	2277	165
165	1129	1129	877	6330	1091	397	124	955	877	148	124	148
1091	2285	285	2124	2295	2124	665	2299	983	2870	2299	165	877
2277	2317	877	124	2285	2277	885	1129	950	202	2277	1129	1129
1129	3421	2285	1129	324	2317	891	2317	958	2126	113	117	2285

Consequently, different algorithms had different advantages for different networks. However, the proposed GLI algorithm performed generally the best among all algorithms on the above-presented three networks, having the most obvious advantages.

(4) Node ranking comparison of 11 algorithms

**Figure 4:**
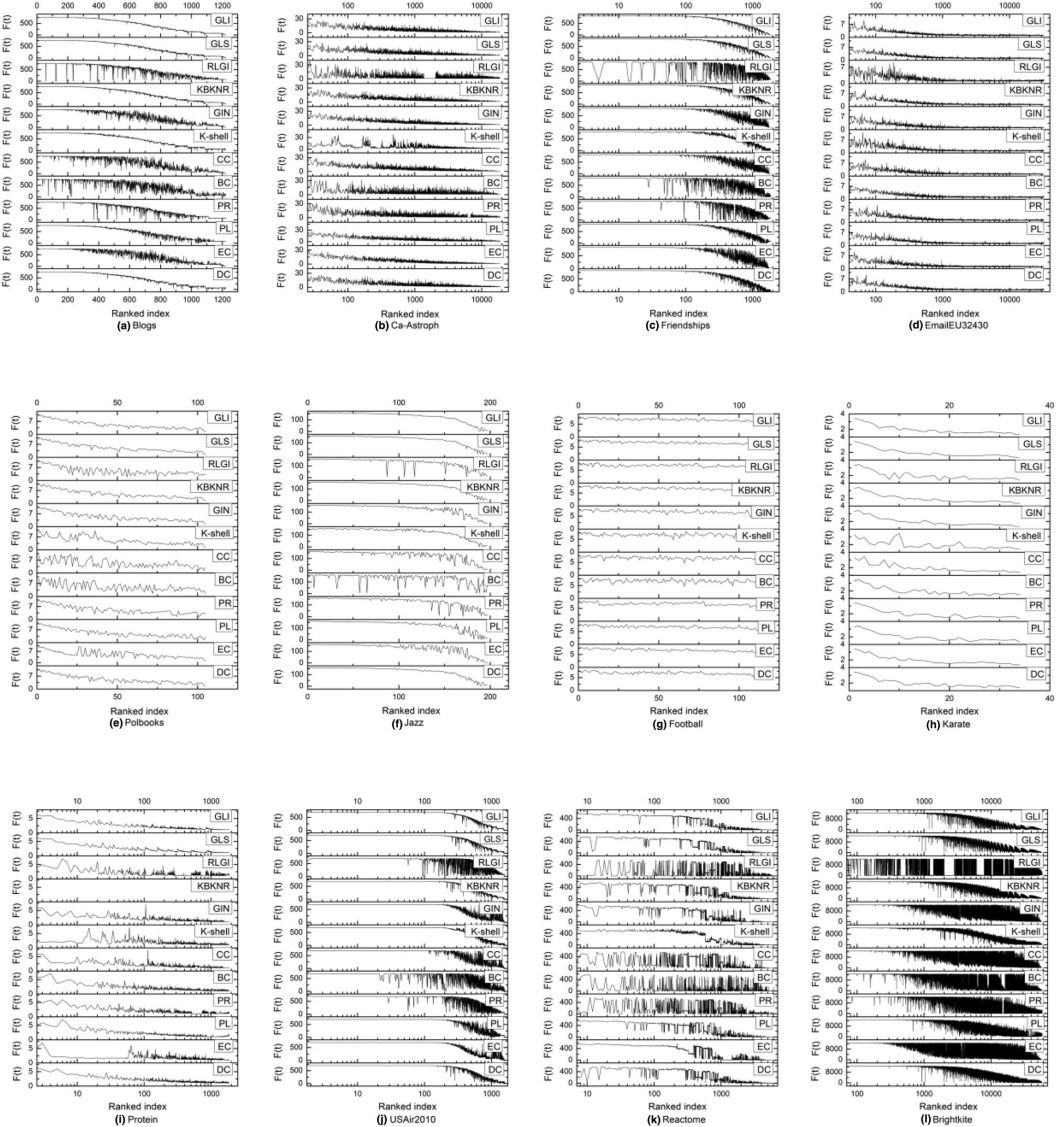
Function F(t) indicates the number of infected nodes in the network at time *t*. For the purpose of reliability of the results, the average result of many iterations is presented as the infection node number value. Due to the large data size of the Brightkite, EmailEU32430 and Ca-Astroph networks, for these networks, the number of iterations was set to 100, and for the other networks, the number of iterations was set to 1000.

The above section analyzes the results of the top-15 key nodes identified by different algorithms and the SIR model in the Karate, Jazz, and Ca-Astroph networks. This section analyzes the sorting results of all nodes in each of the twelve networks. First, the infection values of nodes were calculated in the SIR model. Next, the nodes were arranged in descending order according to their importance values obtained by each algorithm. Then, the sequence of infection values was obtained from the node ranking results. It should be noted that if the ranking results of the algorithm were consistent with the results of the SIR model, a curve with a smooth downward trend from left to right would be formed. The results of a single node denoted as a seed node according to its infection value obtained by different algorithms are presented in Fig. 4, where the abscissa represents the number of infected nodes in the network obtained by each of the algorithms, and the ordinate represents the number of nodes infected and recovered at time *t*.

In Fig. 4, the data of the Polbooks, Jazz, Football, and Karate networks, which were small networks, are displayed on the linear scale; for the remaining eight networks, which had a large number of nodes, the data are displayed on the logarithm scale, focusing on the most influential nodes. As shown in Fig. 4, for the Blogs network, the result of the GLI algorithm showed an overall smooth decreasing trend, with the least number of peaks among all the algorithms. For the Ca-Astroph, Friendships, Brightkite, Reactome and USAir2010 networks, the results of the GLI algorithm had a few peaks, indicating that individual nodes were biased, but the proposed GLS algorithm’s results had the best effect among all the algorithms. For the EmailEU32430 network, the GLI, GLS, KBKNR, and K-shell algorithms performed well, but the curve decline of the results of the KBKNR and K-shell algorithms was reduced, the proposed GLS algorithm’s results fluctuated less and had the best effect among all the algorithms. Further, for the Polbooks network, the proposed GLI algorithm’s results fluctuated less and had the best effect among all the algorithms. For the Jazz network, the right part of the curve formed by the GLI algorithm had the least fluctuation and the best effect among all the algorithms. However, for the protein network, the KBKNR algorithm could not run, so its curve is not shown in Fig. 4, and among the remaining algorithms, the GLI algorithm achieved the best results. Therefore, the GLI method performed the best among the ten networks on the Blogs, Ca-Astroph, Friendships, EmailEU32430, Polbooks, Jazz, Protein, USAir2010, Reactomeand and Brightkite networks. The data curves in the stacked map showed a smooth downward trend, which was consistent with the SIR model results. For the Football network, due to the small difference in the degree value between the nodes, the curves of all algorithms showed certain fluctuations. The fluctuations of the KBKNR and EC algorithms were small, and their effect was relatively good. In the Karate network, except for the obvious curve fluctuations of the K-shell, CC, BC, and PR algorithms, the other algorithms showed a smooth downward trend, with a slight difference.

Consequently, the proposed GLI algorithm performed the best among all the algorithms on most networks, having similar results as the SIR model. Thus, the proposed algorithm could accurately identify key nodes in the networks.

#### Experimental results comparison with the SI model

To analyze the performance of the proposed algorithm further, the SI model was used to evaluate the key nodes identified by different algorithms. Due to limited space, only the Kendall values obtained by the algorithms are presented in this section.

The value of the infectious probability α plays an important role in the experiment. The infected rate is (1/2)^*θ*^(here we set *θ* = 3)^55,57^.

**Figure 5:**
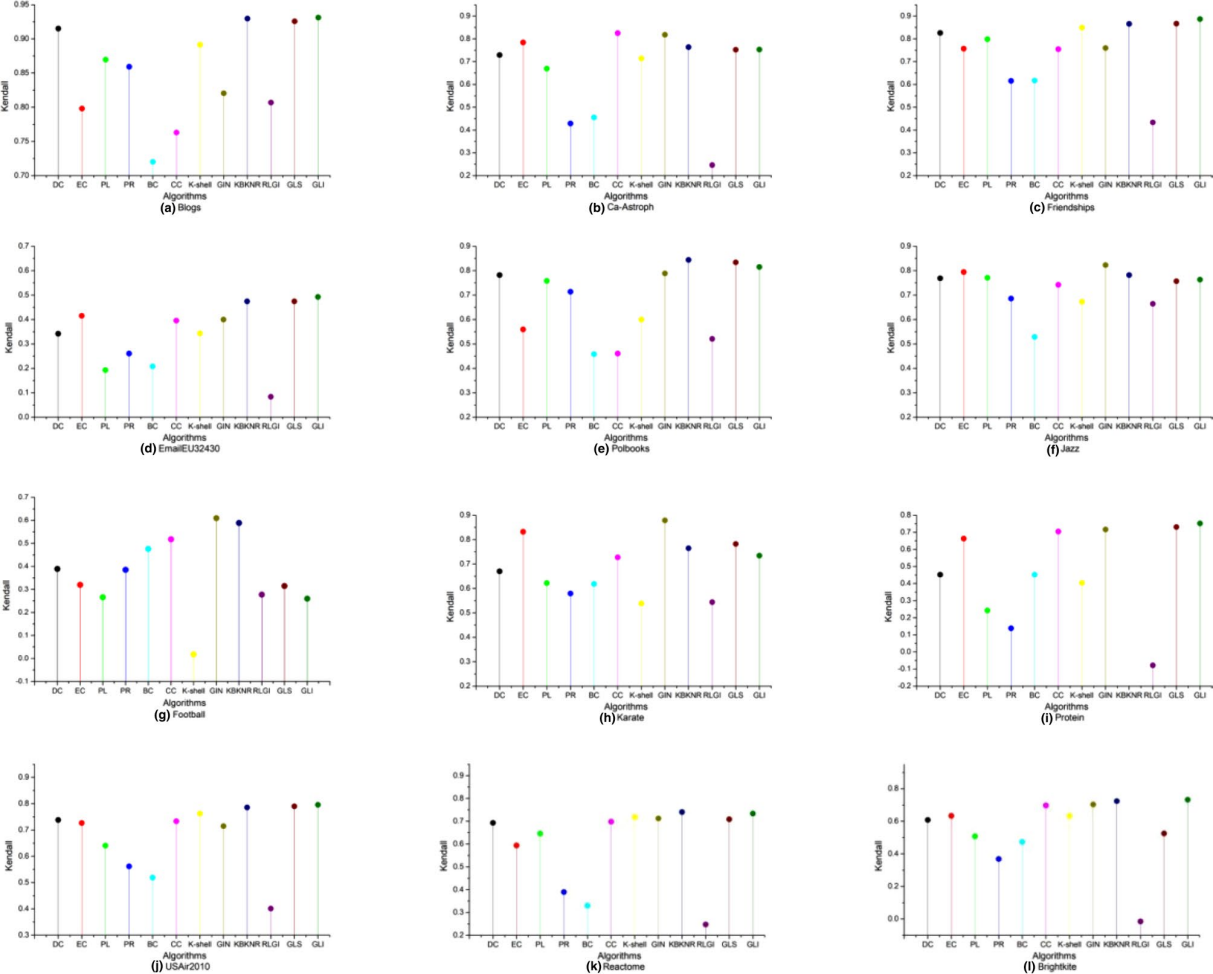
Kendall τ values of different algorithms compared with the SI model result on 12 networks. The value of the infectious probability a is 0.125.

The Kendall values obtained by different algorithms for different networks are presented in Fig. 5.

As shown in Fig. 5, the Kendall *τ* value of the GLI algorithm was higher among all the algorithms for the Blogs, Friendships, USAir2010, Protein, Brightkite and EmailEU32430 networks. In the Jazz, Football and Karate networks, the Kendall *τ* values of the GIN algorithm were superior to the other algorithms. In the Polbooks and Reactome networks, the Kendall *τ* values of the KBKNR algorithm were highest. Only in the Ca-Astroph network, the Kendall *τ* values of the CC algorithm were highest. Consequently, the proposed GLI algorithm performed the best among all the algorithms on most networks.

### Infection capability of the top 15 nodes

In order to validate the effectiveness of the GLI algorithm, we have calculated the infection ability of the top 15 nodes of the GLI and other algorithms in the SIR model. In the experiment, the infection probability α has been set to 0.01, and the recovery probability β has been set to one, the time step has been set from 1 to 30, and the number of iterations has been set as 1000.

**Figure 6:**
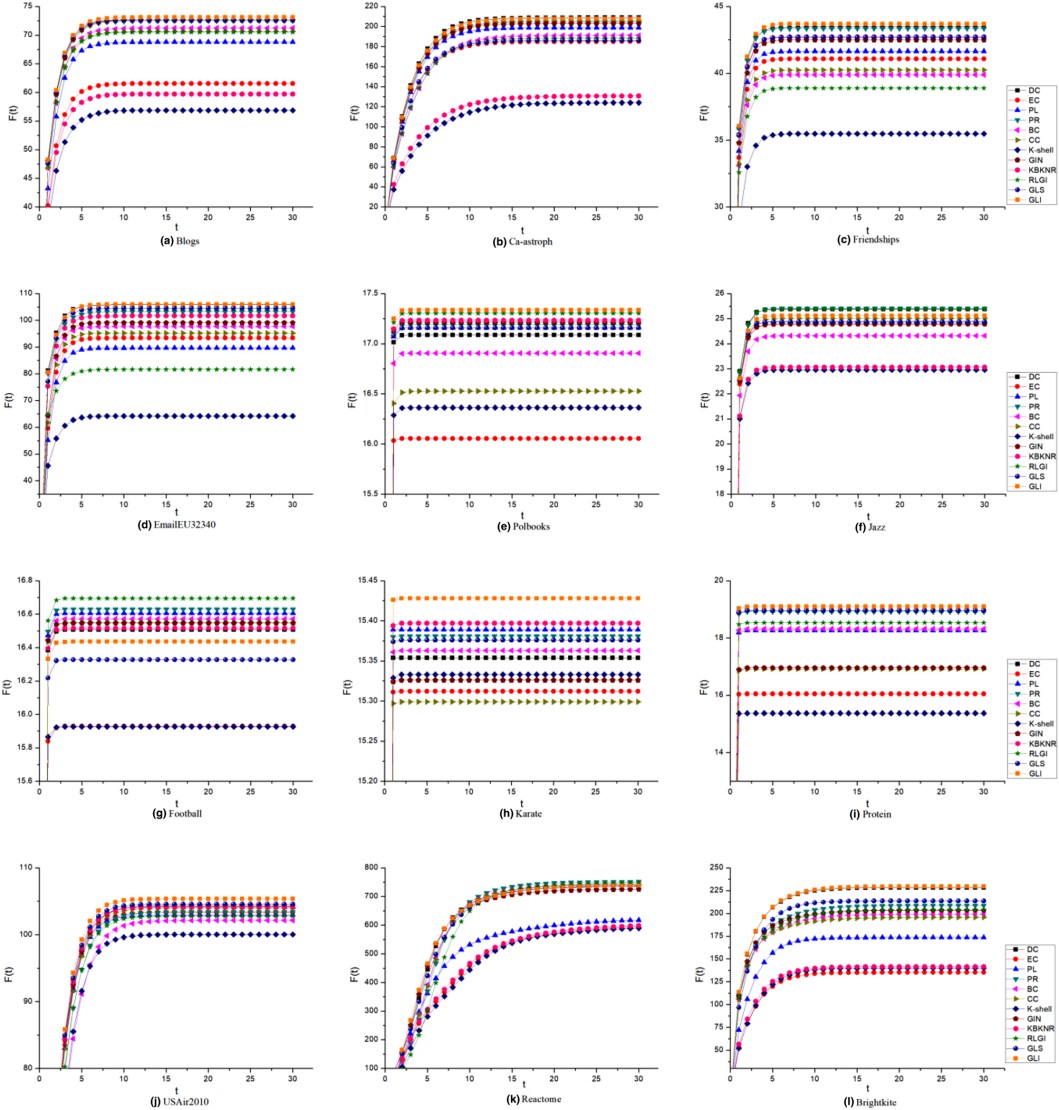
The infection capability of top 15 nodes in the ranking of twelve different algorithms. F(t) represents the number of nodes that were infected by these top 15 nodes at time t.

As shown in Fig. 6, the number of infected nodes F(t) increased with the increasing time step t, and finally it reached a stable value at time step t = 10. This indicated that the top 15 influential nodes effectively infected other nodes in a short time. In the eight networks, namely the Blogs, Friendships, EmailEU32430, Polbooks, Karate, Protein, USAir2010 and Brightkite networks, the top 15 nodes of the GLI algorithm had the strongest infection ability. In the Ca-Astroph network, the infection ability of the top 15 nodes of GLI algorithm and DC algorithm were similar and better than other algorithms. In the Jazz and Reactome networks, the top 15 nodes of the RGLI algorithm and the PR algorithm had similar infection abilities, with distinct advantages over the GLI algorithm. But the GLI algorithm performed better than the other algorithms. In the football network, the effect of the GLI algorithm was general. Therefore, the top 15 nodes which selected by the GLI algorithm were stronger than other algorithms in the majority of networks. The result further demonstrated the effectiveness and accuracy of the proposed algorithm.

### Time complexity analysis

The time complexity analysis was performed considering the procedure performed by the proposed GLI algorithm, including three stages. The temporal complexity analysis results are described below.

First, the network was stored in the logical form of an N $\times$ N matrix, and the computation degree needed to go through the other (*n* − 1) nodes, and the time complexity was *O*(*n*^2^). Further, the time complexity of calculating the K-shell value was *O*(n*log*n*). Next, the given value of the adjacent node was calculated, and this process involved the adjacent node; the time complexity of this process was *O*(n*(*n − *1)) when the network was a complete network.

The total time complexity was calculated as the maximum of the above three time complexity values, and the time complexity of the proposed GLI algorithm was *O*(*n*^2^).

The source code is available online at: https://github.com/hhf602/GLI-Code.

## Conclusion

This paper proposes an efficient and accurate algorithm for key node identification in a complex network named the GLI algorithm. The GLI algorithm first calculates the *Ks* value of a network node, which is expressed as a global influence. Then, the local influence is obtained considering the node degree, the adjacent node’s node degree value, and the adjacent node’s *Ks* value and introducing a similarity coefficient between the adjacent nodes. Finally, the node influence is calculated based on the global and local influence results. The proposed algorithm is verified by experiments. It is compared with the other related algorithms using the results of the SIR and SI models as the evaluation index. Based on the experimental results of the nine algorithms on ten networks, the proposed GLI method performs better than the other algorithms.

The main reason why the GLI algorithm achieves better results than the other algorithms is that in the proposed algorithm, the network nodes are ranked by the K-shell algorithm, and then the ranking result of the K-shell algorithm is refined according to the local influence of the nodes. This enhances the differentiation of node influence and solves the problem of hierarchical coarse-graining of the K-shell algorithm.

However, the proposed algorithm considers only information of adjacent nodes but not the information of secondary or tertiary neighbors of a node when calculating the local information influence contribution. Therefore, multiple influencing factors could be considered in further in-depth research.

## Supplementary Information

Supplementary Information.

## Data Availability

The datasets analysed during the current study are available in the ScienceDB repository, https://www.scidb.cn/anonymous/WmpxbUF6.
