# Supplementary material for: Exploring influential nodes using global and local information
Source: Sci Rep. 2022 Dec 29;12:22506. doi: 10.1038/s41598-022-26984-4 (PMC9800360; doi:10.1038/s41598-022-26984-4)
Supplement: Supplementary file 1 — Supplementary Information. [file 41598_2022_26984_MOESM1_ESM.pdf]

```
@author: pdsu
import networkx as nx
import pylab
import random
path = 'CA-GrQc.txt'
def simjkd(u, v):
        set_v = set( G.neighbors(v))
        set_v.add(v)
        set_u = set( G.neighbors(u))
        set_u.add(u)
        jac = len(set_v & set_u) * 1.0 / len(set_v | set_u)
        return jac
G = nx.Graph()
with open(path) as file:
        for line in file:
                head, tail = [int(x) for x in line.split()]
                G.add_edge(head, tail)
nums = G.number_of_nodes()
G.remove_edges_from(nx.selfloop_edges(G))
k_shell=nx.core_number(G)
print('k_shell :',k_shell)
maxKshell=max(k_shell.values())
minKshell=min(k_shell.values())
maxD=max(dict(G.degree()).values())
print("maxD",maxD)
def getCountKshell(G):
        node = G.nodes()
        print("node=",node)
        ks_classfity = [dict(g) for k, g in groupby(sorted(nx.core_number(G).items(),
key=by_value), by_value)]
        print(ks_classfity)
        dicts = {}
        for index in node:
                list = []
                print("index=",index)
                for ks_value in ks_classfity:
                        dictss = {}
                        for k,v in ks_value.items():
                                if k == index:
                                        continue
                                dictss[k] = nx.shortest_path_length(G,source=index,target=k)
                        list.append(dictss)
                dicts[index] = list
        return dicts
```

```
d=[]
res={}
for nodev in G.nodes():
    value=0
    for nodeu in G.neighbors(nodev):
        xs=simjkd(nodev,nodeu)
        value+=xs*(G.degree(nodeu))+k_shell[nodeu]
    res[nodev]=value/maxD+G.degree(nodev)+k_shell[nodev]
print(res)
for key in res.keys():
    rest = res[key]
    d.append((key, rest))
sortNum = sorted(d, key=lambda x: x[1], reverse=True)
nodelist=[]
sortNum = sorted(res.items(), key=lambda x: x[1], reverse=True)
for key in sortNum:
    nodelist.append(key.__getitem__(0))
print(nodelist)
f =open('outputdata\\hu_'+path, "w+")
for key,val in sortNum:
   f.write(str(key)+'\t'+str(val)+"\n")
f.close()
nodelist1=[]
sortNum1 = sorted(res.items(), key=lambda x: x[0], reverse=False)
for key in sortNum1:
    nodelist1.append(key.__getitem__(0))
print(nodelist1)
f =open('outputdata\\hu_1'+path, "w+")
for key,val in sortNum1:
   f.write(str(key)+'\t'+str(val)+"\n")
f.close()
```
